# The role of metacognition in human social interactions

**DOI:** 10.1098/rstb.2012.0123

**Published:** 2012-08-05

**Authors:** Chris D. Frith

**Affiliations:** 1 University College London, Aarhus, Denmark; 2 Aarhus University, Aarhus, Denmark; 3 All Souls College, Oxford, UK

**Keywords:** metacognition, mentalizing, we-mode, monitoring, control, prefrontal cortex

## Abstract

Metacognition concerns the processes by which we monitor and control our own cognitive processes. It can also be applied to others, in which case it is known as mentalizing. Both kinds of metacognition have implicit and explicit forms, where implicit means automatic and without awareness. Implicit metacognition enables us to adopt a we-mode, through which we automatically take account of the knowledge and intentions of others. Adoption of this mode enhances joint action. Explicit metacognition enables us to reflect on and justify our behaviour to others. However, access to the underlying processes is very limited for both self and others and our reports on our own and others' intentions can be very inaccurate. On the other hand, recent experiments have shown that, through discussions of our perceptual experiences with others, we can detect sensory signals more accurately, even in the absence of objective feedback. Through our willingness to discuss with others the reasons for our actions and perceptions, we overcome our lack of direct access to the underlying cognitive processes. This creates the potential for us to build more accurate accounts of the world and of ourselves. I suggest, therefore, that explicit metacognition is a uniquely human ability that has evolved through its enhancement of collaborative decision-making.

## 1. Introduction

The remarkable dominance of human beings over other creatures and their ability to control physical forces is a result, in part, of their ability to work together in groups to achieve more than the total work of the individuals involved. In this paper, I will argue that this outstanding feature of human social life depends critically on metacognition. First, therefore, I will briefly outline what I mean by metacognition and make a distinction between the implicit and the explicit forms of metacognition. I will then discuss the role of mentalizing in social interactions, pointing out that this kind of metacognition also has an implicit and an explicit form. Finally, I will show in what way explicit metacognition enables the kinds of group activity that humans are so good at and why explicit metacognition should be considered a uniquely human ability.

## 2. Metacognition and mentalizing

### (a) Metacognition and self-monitoring

The term metacognition refers to the cognitive processes involved in thinking about thinking. Metacognitive processes were first discussed by psychologists interested in strategies for improving learning and memory [1]. These are the processes by which people reflect on their memories (monitoring) and use the knowledge so acquired to regulate these processes (control) [2]. One consequence of monitoring memory might be an experience of the ‘tip-of-the-tongue’ state, in which we feel that we know the answer, even though we are unable to recall it at that moment. A strategy to regulate the retrieval of the word is to deliberately and systematically go through the alphabet testing possible target words beginning with each letter.

More recently, related metacognitive processes of monitoring and control have been studied in signal detection tasks and in reaction time tasks [3]. In the studies of reaction time, the emphasis has been on error detection (monitoring) and on changes in behaviour that occur after an error has been detected (control). For example, after an error, reaction times often increase, reflecting the adoption of a more cautious strategy. However, these studies show that post-error corrections and changes in strategy can occur automatically and quite independently of explicit error detection (for a review see [4]). This dissociation was observed strikingly in a study of skilled typists in which the experimenters supplied false visual feedback by correcting some of the errors the typists had made and inserting errors that they had not made [5]. The typists slowed down after corrected errors and did not slow down after inserted errors, showing that this outcome of self-monitoring was driven by real errors and was not affected by the false feedback. Nevertheless, many of the typists accepted responsibility for the inserted errors and were unaware of the errors that had been corrected for them.

These results reveal two aspects of metacognition, which are of critical importance to my thesis in this paper. First, there seem to be two forms of self-monitoring. There is an explicit form, which is slow and deliberate, while there is also an implicit form, which is rapid, automatic and can occur without awareness. The question remains open as to whether this implicit form of self-monitoring should even be called metacognition (see [6] for a discussion of this question). Second, the explicit form of self-monitoring, as we shall see, is highly susceptible to error.

### (b) The limitations of explicit metacognition

I assume that explicit metacognition is concerned with generating reportable knowledge about the processes underlying our behaviour. However, conscious access to these processes seems to be severely limited. This was the case for the skilled typists mentioned earlier and has been observed in many other experiments [7]. These studies confirm the general principle, outlined in the review by Nisbett & Wilson [8], that we have little or no direct conscious access to higher order cognitive processes. We may have access to the outcomes of these processes, but, through introspection, we get very little idea as to how these outcomes are achieved.

In some circumstances, we have rather limited access even to the outcomes of decision-making processes. An example comes from an experiment in which people were asked to choose between two kinds of jam. Having chosen, they were re-presented with their chosen brand and asked to try it again and explain why they had chosen it [9]. However, on some occasions, a trick was used so that participants were presented with the jam they had rejected. On more than half of these occasions, the switch was not noticed and people justified a choice they had not actually made (change blindness see also [10]). In this scenario, people seem more concerned with explaining and justifying their decision-making process rather than with checking what they actually decided. This gives us an important clue to the value of explicit metacognition. At the conclusion of this paper, I will suggest that it is this willingness to make metacognitive reports on the causes of behaviour, whether or not they reflect the true state of affairs, that gives humans their dramatic advantage in group activities.

### (c) Metacognition and mentalizing

Mentalizing (aka Theory of Mind) refers to our ability to take account of the mental states of others (monitoring) and to use this information to predict behaviour (control) [11]. The development of this ability has been studied extensively using false belief tasks [12]. To pass such tasks, children have to recognize that someone's behaviour will be determined by that person's belief, even when this belief is clearly false. So, for example, the protagonist will look for his chocolate where he believes it to be, and not where it actually is. This ability is robustly observed to emerge between the ages of 4 and 6 (reviewed in [13]). At this age, children can justify the behaviour of the protagonist, and their own interpretation of this behaviour in terms of knowledge and belief: ‘he looked in the cupboard, because that's where he put the chocolate and he didn't know his mother had moved it to the fridge’. At around the same age, children can also justify their own behaviour in terms of their knowledge and beliefs (see [13], p. 665 and [14]): ‘I looked in the cupboard because I didn't know the chocolate had been moved’. Performance of this task requires explicit mentalizing.

I consider that this understanding of behaviours in terms of beliefs and desires is an example of explicit metacognition, whether it is applied to the self or to others. In both cases, we are reporting knowledge that we believe we have about the putative reasons underlying behaviour. We understand and justify behaviour, whether our own or others’, as the logical outcome of certain beliefs and desires.

## 3. Mentalizing and joint action

The role of mentalizing in deception and Machiavellian behaviour is often emphasized because the ability to deceive is a reliable marker of mentalizing ability [15,16]. However, mentalizing is also crucial for many aspects of non-deceptive and collaborative behaviour. For successful joint action, we need to take account of other peoples' knowledge, goals and values [17], and there is evidence that the ‘collective intelligence’ of human groups is higher when the group members have greater social sensitivity [18]. But it does not follow that explicit metacognition is essential for joint action.

There is now considerable evidence from studies of joint attention and joint action that suggests that there is an implicit form of mentalizing through which we can take account of the mental states of others without being able to provide justifications [19]. As we have already seen in the case of reaction-time tasks, implicit processes are rapid, automatic and occur without awareness. In general, automatic processes generate behaviour in an unwilled and unreasoned way [20]. Such processes also enable us to take account of the mental states of others. Explicit processes in contrast have deliberate and reasoned content, even when these reasons are not based on reality, as in the change-blindness tasks [9].

### (a) Implicit representation of the goals of others

In an innovative series of studies, Sebanz *et al*. [21] have shown that people automatically represent the goals of the person they are working with. The first of these studies capitalized on spatial compatibility effects in a reaction-time task (the *Simon effect*). The imperative signal was colour: press the left button for a red stimulus and the right button for a green stimulus. However, the stimuli also varied in spatial location, which could be congruent or incongruent with the required response. Thus, the response was congruent when the red stimulus was left oriented and incongruent when the red stimulus was right oriented. When the task was performed by a single individual as a two-choice reaction-time task, there was a strong effect of congruence, that is, congruent responses were made faster than incongruent ones. When, however, the task was performed as a go/no-go task, so that the participant had only to press the left button to red stimuli, the congruency effect disappeared. The innovative condition involved bringing in a second participant to perform the other half of the go/no-go task, i.e. to press the right button to the green stimulus. In this context, even though the original participant was still performing the identical go/no-go task, the congruency effect returned and spatially incongruent responses were slowed. This effect has been confirmed and elaborated in a number of subsequent studies [22]. The effect suggests that, when performing a task alongside someone else, one cannot help but represent also the stimulus–response requirements of the task the other person is doing. We know that this representation of the goals of others occurs automatically, since it is detrimental to the performance of an on-going task. In other words, the rational operator would choose not to represent the other person's representation of the task.

### (b) Implicit representation of the knowledge of others

Having a different spatial view-point can create incongruence of knowledge, since what one person can see often differs from what another person can see. Many studies have demonstrated an effect of such incongruence (see [23] for a review). For example, given that I can see everything in a room (bird's eye view), I take longer to report what another can see (e.g. number of pictures) if it is different from what I can see. This is due to an egocentric bias towards my own point of view [24]. Samson *et al*. [23] report a novel twist on this phenomenon, which shows a detrimental effect even when there is no need to represent the other person's view-point. The participants were never asked how many pictures the other person could see, but only how many they could see. Nevertheless, the mere presence of another person in the room with different knowledge slowed down this egocentric response. This process is automatic, since the result was shown to be unaffected by cognitive load [25]. This observation shows that we cannot help taking account of the knowledge of others when it is different from our own.

### (c) Implicit representation of the beliefs of others

At around 5 years of age, children develop an explicit form of mentalizing and can explain the relationships between beliefs and behaviour. However, there is an implicit form of mentalizing, which is already in place before 12 months of age and remains present even in adults. This form is revealed by the use of non-verbal measures such as looking time and reaction time that are also affected by discrepancies between the beliefs of self and others.

For example, infants of seven months as well as adults were shown a scenario in which a ball hid behind a screen [26]. Under some conditions, the ball then emerged again and left the scene. Finally, the screen was raised to reveal the ball or an empty space. The infant's looking time was used as a measure of surprise. If the ball was unexpectedly revealed to be behind the screen, the infants looked longer. Under the critical conditions, another observer, *a Smurf*, was also present. This observer would be present when the ball hid behind the screen, but might be absent when the ball emerged again and left the scene. When this observer returned, he would have the false belief that the ball was still behind the screen. The presence of this observer with a false belief influenced the behaviour of the infants. In the presence of a Smurf who falsely believed that the ball was still present, they were not so surprised (in terms of shorter looking time) by the appearance of the ball even though they had seen it leave. The same effect was shown by adult participants, for whom reaction time to report the presence of the ball was used, rather than looking time.

These observations suggest that adults and infants automatically take account of the beliefs of others when these beliefs are different from their own (see [27] for a review of these studies).

### (d) An implicit we-mode for joint action

In the tasks described earlier, automatically taking account of the knowledge and intentions of others made individual performance worse. However, for successful joint action, it pays for us to take account of our partners' goals, knowledge and beliefs. Ideally, these need to be shared in such a way that everyone in the partnership operates in the *we-mode* rather than in the *I-mode* [28]. The automatic processes revealed by the studies I have just reviewed would provide a mechanism by which the adoption of a we-mode could be advantageous.

I suggest that the we-mode significantly changes the value or salience of stimuli in the group field. In order to interact successfully with the world, we need to restrict our attention to the objects and the actions most relevant to our current goals. This can be achieved by representing objects and actions in a *saliency map* [29] or *value map* [30]. In this map, objects relevant to current goals have higher saliency values and more readily elicit attention. However, the value of the objects will be modified by the extent to which actions, such as grasping, can be performed on them. So, for example, objects that are out of reach will have lower saliency values. When I am engaged in joint action, or even in the mere presence of other people, my saliency map is modified so that the value of the various objects reflects something approximating to the average values of the group derived from my implicit estimates of the goals, knowledge and beliefs of the other group members. Thus, for example, a relevant object that was within the reach of someone else in the group would have a high value even though it was outside my reach. A further prediction would be that relevant objects that other people could not see would have lower values even though I could see them. This may relate to the biased pooling of information observed by Stasser & Titus [31]. Group discussions are biased towards information that group members already held in common before discussions begin. The group does not gain full advantage from the pooling of un-shared information. I propose that it is the adoption of the we-mode that causes this automatic adjustment of our view of the world.

If this account of implicit mentalizing is correct, it could perhaps be argued that it should not be called mentalizing. The knowledge and desires of others are not represented as mental states. Rather, the mental states of others are taken into account automatically by altering the saliency and values of objects and actions that are at the focus of joint attention. People behave ‘as if they were mentalizing’ [32].

## 4. Explicit metacognition about actions of the self

At about 4 years, after the emergence of metacognition, children can reflect on the relationship between knowledge and action. This kind of reflection is an example of explicit metacognition, but what does this ability add to the implicit processes discussed earlier?

Reflecting on our actions is a major feature of human mental life. We think about which acts to perform and when to perform them. Such introspection suggests that explicit metacognition determines our behaviour, but the way actions feel to us is not a good guide to how they are controlled. For example, when participants were asked to lift a finger *whenever they felt the urge to do so* and to indicate the time at which this urge occurred, the time of the urge was found to occur approximately 300 ms *after* the first appearance of the changes in brain activity associated with a voluntary action [33]. These results are also consistent with those of some studies showing that reaching and grasping responses can be initiated automatically, with awareness occurring hundreds of milliseconds after initiation ([34], see [35] for a review). What is the relevance of these experiences that occur after the initiation of an action?

An important clue is provided by the work of Haggard *et al*. [36] showing that actions and their consequences are experienced as closer together in subjective time than in objective time. Such *intentional binding* does not occur when the action is involuntary [36]. These results suggest that reflection on action is not necessary for the production of action, but may be critical to experience of outcomes, following actions, as intended or accidental. The phenomenon of intentional binding creates our experience of agency and also creates a sense of responsibility [37]. Such experiences play a crucial role in human social interactions.

## 5. The social function of explicit mentalizing about action

In this paper, I will suggest that the major, if not the only, function of explicit metacognition is to enhance social interactions. To justify this proposal, I should make it clear that I understand metacognition as allowing us to communicate our thoughts and reflections to others. Such communication need not depend on language. It could also be carried by gestures [38]. I propose that the ability to reflect on and report our actions and experiences can improve collaboration over and above the we-mode of implicit mentalizing. It allows us to optimize the sharing of resources and the sharing of information. At the same time, social interactions enhance metacognition. Through discussions with others, we improve our ability to give a more accurate report on the reasons for our actions and experiences.

### (a) Agency, responsibility and altruistic punishment

Our experience of agency carries with it a sense of responsibility [37,39]. We experience a marked difference between intended outcomes and outcomes that occur by accident. We also make this distinction for the acts of others and respond to errors made by others in the same way that we respond to errors made by ourselves [40]. We feel more regret for ourselves and apply more blame to others when a bad outcome is the result of an intentional act rather than an accident [41].

I suggest that these feelings have a fundamental role in collaborations concerned with the sharing of resources. In a *common goods game*, the group as a whole benefits from individual players collaborating by putting money into a pool, which is then augmented and shared out among all the players. However, collaboration and hence group benefit is diminished by the appearance of *free riders*, that is unfair players who put in no money themselves but receive the group benefits. Free riding can be reduced and collaboration enhanced by permitting *altruistic punishment* [42]. This punishment takes the form of a fine and is altruistic in the sense that the punisher has to pay for the punishment to be applied. As would be expected, punishment is applied to unfair players with greater punishment for more unfair play [41]. However, in a study by Singer, there were two kinds of players: those who had a free hand in making their decisions about how much money to donate and those who had no free hand and simply followed written instructions. Even though the monetary loss was the same, the people who were not responsible for their actions were not punished. This result suggests that our experience of agency and of responsibility for actions has a critical role in maintaining cooperation and group benefit.

### (b) Discussions of the nature of action can change behaviour

As reviewed earlier, introspection of our actions can be fragile and erroneous. However, we can learn about the nature of action and decision-making through observing others and hearing the justifications they present for their actions. Indeed, there is evidence that we are more accurate in recognizing the causes of the behaviour of others than we are at recognizing the causes of our own behaviour [43]. Therefore, our understanding of our own behaviour is likely to benefit from the comments of others.

Discussions of the basis of actions can alter our experience and can change our behaviour. For example, Vohs & Schooler [44] told one group of students that ‘most scientists now recognize that free will is an illusion’. On a subsequent arithmetic test, these students were more likely to cheat than a group who had not been told anything about free will. I suggest that the statement that free will is an illusion had changed their experience of and attitude towards their own actions. First, their sense of agency and associated responsibility was reduced: cheating would be less deserving of punishment. Second, they might believe, probably erroneously [45], that, without the deliberate control exerted by free will, they could not avoid releasing their basically selfish nature. So how could they resist the option of cheating?

A second example comes from a study of will power. People who have had to resist temptation, e.g. eating the radishes rather than the chocolates in front of them, subsequently show less persistence on a variety of tasks [46]. But how much is this effect due to our understanding of the nature of will power? Job *et al*. [47] in a series of experiments showed that peoples' beliefs about the nature of will power affect their behaviour and that these beliefs and behaviour could be manipulated. People who had been told that will power could be depleted by effort showed less persistence after exerting their will. But people who had been told that will power could be strengthened by practice showed more persistence.

### (c) Metacognition creates beliefs about action that affect our behaviour

We develop beliefs about the nature of action and how best to make decisions through introspection and through our attempts, and those of others, to justify our behaviour. These beliefs alter our behaviour, perhaps through modification of the balance between the many competing processes that determine decisions. Since these individual beliefs are developed through social interaction, they are likely to reflect beliefs that are common to a group. In the long run, cultural norms concerning agency and the appropriate way to make decisions will emerge. In the even longer run, through their effects on behaviour, these beliefs are likely to evolve towards those that optimize the outcomes of decisions. My intuition is that, after such evolution, the beliefs will reflect more closely the cognitive processes underlying decision-making. Through discussion with others, we can overcome the fragility of our introspection and learn to experience ourselves better.

## 6. The social function of explicit introspection about sensation

We have recently shown that two people working together to detect a subtle visual signal can do better than the best one working on his own. In this task, participants must decide in which of two intervals the signal occurred. In each interval, six black-and-white striped (Gabor) patches are presented arranged in a circle. In one of the two intervals, one of the patches (the odd-ball) has a contrast slightly different from that of the other five standard patches. Participants must decide in which interval this odd-ball occurred. Performance on this task can be measured very precisely in terms of the psychophysical curve relating the probability of interval choice to the difference in contrast between the oddball and the standards. The steeper this curve, the better the performance. Participants saw the stimuli and then reported individually whether the oddball had occurred in the first or in the second interval. If they disagreed, they had a free discussion and came up with a joint decision. In terms of the slope of the psychometric function, the group decisions were significantly better than decisions of the better of the two partners (group advantage). For this task, two heads were better than one [48].

To better understand this result, we developed a computational model of how information might be aggregated across the two partners. This model was based on previous work on the aggregation of information across two senses (e.g. vision and touch) within one participant [49]. In this case, the senses are integrated in a statistically optimal fashion with greater weight being given to the less noisy sense (Bayesian inference [50]). For such optimum integration to occur when two people share information, they would also need to take account of how confident each was in what they had just seen and put more weight on the more confident partner. We found that the optimum performance predicted by a *weighted confidence-sharing* model gave a very good fit to our data.

To achieve this optimum performance, the partners would need to report to each other their confidence on each trial. And indeed it was the case that optimum performance could be achieved only when the partners were permitted a discussion before submitting a joint decision.

A detailed analysis of the linguistic content of the discussions revealed that, during the course of the experiment, each pair developed a unique set of verbal descriptions providing a scale for communicating their confidence [51]. Here are two examples (translated from the Danish): pair 21 (*sure, almost sure, a little uncertain, not sure, very unsure, totally unsure*), pair 43 (*saw it well, think I saw it, couldn't see, didn't see anything, only saw a blank*). The more rapidly a pair developed and used a small set of such phrases for communicating confidence, the greater their group advantage.

As these observations indicate, our subjects needed time to learn how to achieve the advantage of working together on this task. Typically, learning is guided by some kind of feedback or outcome signal and, in our first experiment, subjects were told, after each trial, whether their joint response was correct and also which partner's initial individual response had been correct. However, in subsequent experiments [52], this feedback was sometimes eliminated from the experimental paradigm. These experiments revealed that feedback was neither necessary nor sufficient for the achievement of a group advantage. No advantage was obtained if feedback was given in the absence of discussion, while advantage was obtained when there was discussion, but no feedback. Group advantage was achieved more slowly in the absence of feedback, but, in the second of two sessions of 128 trials, the group advantage for the partners who got no feedback was identical to that of partners who did.

These results show that, when two people discussed their experiences, objective external feedback was not needed to acquire an accurate perception of the world. Apparently, at least when shared with others, subjective experience is sufficient for forming reliable beliefs about the world. This sharing of experiences depends on explicit metacognition.

### (a) Group advantages depend on relative ability and the mode of communication

Collaboration on the signal detection task is not always advantageous. If partners have very different abilities, the weighted confidence-sharing model makes the prediction that the pair will perform worse than the better partner. This was confirmed in further experiments [48,53] in which noise was added to the signals presented to one of the two partners to lower his perceptual ability. This loss of advantage occurred even when the noise was always added to the same individual in the pair so that his performance was consistently poor.

This effect critically depended on how the partners communicated their confidence. The effects just described did not occur when confidence was communicated using a non-verbal system supplied by the experimenters [53]. This eliminated the disadvantage of working with a less-competent partner. On the other hand, although the advantage remained when partners had similar levels of competence, this advantage was not as great as that associated with unconstrained verbal communication.

These results show that the strategy of weighted confidence sharing, especially when this is achieved through free discussion, should be used only when partners have similar competence. So why do partners continue to use this strategy when their competence is very different? We have suggested [53] that this problem is the result of the various automatic biases that are known to undermine communication and group decision-making. Because of the *egocentric bias* [54], we assume that our partner is similar to us. Because of the *illusion of transparency* [55], we assume that our internal states are more discernable to others than they really are. Because of the *hidden profile problem* [31], too little weight is put on information that is known only to one member of the pair (i.e. the more competent partner). When the members of the pair are indeed similar on the relevant abilities (and have the same goals), these biases can be an advantage since the weighted confidence-sharing model is optimal. But if the members of the pair are dissimilar, these biases interfere with the adoption of more appropriate strategies, for example, putting much less weight on the advice of the incompetent partner.

We assume that these biases are more pronounced when partners interact using free and direct verbal communication. This is because direct verbal communication is much more likely to move us into the we-mode, in which, as outlined previously, all these biases listed come into play precisely to make us more similar, in terms of knowledge, intentions, etc. to the person we are talking to. When using the non-verbal communication system, we remain more isolated from our partner, in part because this novel system requires greater cognitive effort to convert our feeling of confidence into a communicable spatial form.

### (b) Sharing confidences improves individual performance

We also observed an unexpected by-product of the metacognitive discussions. Since all participants made an individual decision before they made their joint decision, we could examine individual as well as joint perception. Participants engaged in an interaction showed a rapid improvement in individual performance and performed significantly better than participants who performed the same task but did not interact with one another [56]. This result suggests that sharing perceptual experiences through discussions with others is an efficient way of improving our individual perceptual abilities. Whether the effect is related specifically to improvements in metacognitive abilities remains to be explored.

### (c) Metacognition and collaboration

I began this essay by discussing explicit mentalizing, our ability to reflect upon mental states of others, as an example of metacognition. But so far in my discussion of the value of metacognition, the emphasis has been on reflecting on our own mental states. For example, I characterized the discussions of confidence that lead to better group performance as involving a participant reflecting on his confidence and reporting this to his partner. But it is also possible that participants were reflecting on the confidence of their partner as well as on their own confidence. Indeed, it may be the case that we can read the confidence of others more accurately than our own by using additional non-verbal cues such as speed and vigour of behaviour.

There is, however, a key first step for joint action, namely the decision to enter into the collaboration in the first place and, for this, it is critical to reflect on the mental states of others. This decision can be studied in isolation in *coordination games*, in which players benefit by coordinating their behaviour. The best example is the *Stag and Rabbit Hunt* [57]. In this game, the players can hunt either stags or rabbits. If both the players decide to hunt the stag, they will get a large reward. This strategy maximizes payoff. If a player chooses to hunt a rabbit, she will get a small reward whatever the other player does. This strategy minimizes risk. The worst outcome occurs if you decide to hunt the stag and your partner hunts the rabbit. So before you choose to hunt the stag, you must be confident that your partner will collaborate.

Thinking about collaboration is essentially recursive: your partner will collaborate only if she is confident that you will collaborate, your partner will only collaborate if she is confident that you are confident that she will collaborate, etc. [32]. Absolute certainty can never be achieved in this situation [58], but this does not cause problems in the many real-life situations requiring collaboration. For example, if I send Cecilia an email suggesting that we meet for lunch, then I should go to the lunch only if I am confident that she will be there. But how can I be sure she has received my email? She sends a confirmatory email, but how can she be sure that I have received it? In practice, her single confirmation is usually sufficient [59]. We can never be absolutely certain, but, given sufficient confidence in our partner, we will choose to collaborate.

Yoshida *et al*. [60] have developed a computational account of the stag-hunt game. They show that optimum responding can be achieved by estimating the degree of recursion of your partner and that this can be computed on the basis of her choices in a sequential game. Of interest here is the observation that the two key parameters that you need to estimate for optimal play of this game are your partner's degree of recursion and your degree of certainty in this estimate. The certainty of your estimate about your partner is another example of metacognitive knowledge similar to certainty about your perceptions.

## 7. The neural basis of metacognition

The characterization of metacognition in terms of the monitoring and control of cognitive processes links it closely with concepts such as working memory and executive control [61]. Conflict resolution, error correction and emotional regulation all have metacognitive aspects and all are associated with executive control instantiated in prefrontal cortex [62,63]. These observations lack anatomical specificity, although there is a suggestion from such results that prospective judgements are associated with medial prefrontal function, while retrospective judgements are associated with lateral prefrontal function (see [64] for a review of this point and other aspects of the neural basis of metacognition).

Another aspect of metacognition, thinking about mental states, both of self and others, is associated with increased activity in the medial prefrontal cortex (see [65]).

Recent developments in the use of signal detection theory to define metacognitive ability [66] allow more precise measurement of metacognitive accuracy (i.e. knowledge of how accurate one's perception is) as distinct from perceptual accuracy. Studies using such measures have confirmed that frontal cortex has a causal role in supporting metacognition since transcranial magnetic stimulation applied to prefrontal cortex [67] can specifically disrupt metacognitive accuracy while leaving perception intact. Furthermore, prefrontal lesions [68] can also specifically disrupt metacognitive judgements about perception. Greater anatomical specificity is provided by magnetic resonance imaging studies of healthy volunteers. Using signal detection measures, Fleming *et al.* [69] found a positive correlation between the volume of grey matter in Brodmann area 10 (BA10; the most anterior region of the prefrontal cortex) and metacognitive ability (independent of perceptual ability). Using a motor task, Miele *et al*. [70] found that activity in a similar location in BA10 was elicited when participants had to report their degree of agency as opposed to their performance accuracy.

In the future, brain imaging studies of metacognition are likely to follow the lead of decision-making studies in which, rather than tracking objective performance (e.g. metacognitive accuracy), a model-based approach is used [71]. Applying this approach to the study of metacognition, the behaviour of participants would be used, on a trial-by-trial basis, to estimate statistical measures of confidence such as precision. Brain regions could then be identified where activity tracks these estimates of internal representations. As already mentioned, such a computational model has been developed for the stag-hunt game. When playing this game, activity in a medial region of BA10 correlates positively with the current estimated degree of uncertainty about the partner's strategy [72]. Thus, there is convergence from a number of studies in favour of a critical role for the anterior frontal cortex (BA10) in metacognition.

### (a) The function of Brodmann area 10

BA10 occupies the frontal pole of the human brain. It has been suggested [73] that this region has enlarged and undergone changes in connectivity more than any other brain region during the course of hominid evolution. So if there are uniquely human cognitive processes, we might expect to find that this region would be involved. However, in addition to its association with metacognition, activity in this area has also been associated with tasks such as prospective memory and task switching.

There are various interpretations of these studies, but a common theme is that the function of this region is to exert flexible control over cognitive processes. For example, Koechlin *et al*. [74] have suggested that BA10 ‘forms a functional “add-on” at the apex of a hierarchy of prefrontal processes controlling the selection of task sets driving behaviour’ and speculates that this is a uniquely human resource. Along similar lines, Burgess *et al*. [75] suggest that BA10 has a ‘cognitive control function’ especially in situations that require, for example, ‘deliberate concentration on one's thoughts’. These characterizations are closely related to metacognition in its role of monitoring and controlling cognitive processes. As yet, however, I am not aware of any attempts to distinguish the neural bases of implicit and explicit metacognition.

## 8. The evolution of metacognition: what is uniquely human?

If we conceive of metacognition as at the top of a hierarchy of control over cognitive processes, the unique feature of human metacognition might be that it adds another level at the top of this hierarchy of control that allows of a far greater flexibility in planning for the future and in reacting to changing circumstances [74].

Another unique function of human metacognition might concern the content of representations. Humans have the ability to represent stimuli that are not present and actions that have not occurred [38]. The representation of such counterfactuals has a major role in mentalizing and in our experience of agency. When we engage in mentalizing, we assume (both implicitly and explicitly) that other peoples' behaviour is determined, not by the actual state of the world, but by a possible state of the world. Our own behaviour is also determined by possible outcomes. A striking example of this is the effect of anticipated regret. We choose option A to avoid the regret we might feel if we chose option B and it did not work out [76]. However, representation of counterfactuals is required even for more basic learning about actions. For example, we do not just learn the values of actions we perform. We also learn about what would have happened if we had chosen different actions. Recent studies show that in monkeys, as well as in humans, learning occurs for hypothetical pay-offs as well as actual pay-offs [77]. As with the other aspects of metacognition, the frontal pole seems to be the region most specialized for representations about counterfactuals [78]. However, given that this ability is found to a limited extent in monkeys [77], this human ability seems to differ quantitatively rather than qualitatively from that seen in other animals.

I believe that the uniquely human aspect of metacognition concerns its role in enabling fruitful group interactions. For instance, Tomasello and his group (reviewed in [79]) identified the human capacity for collective intentionality as the major factor explaining the social difference between humans and other primates [38]. The concept of collective intentionality captures the idea that humans do not simply act together. Humans working together adopt a group-oriented stance creating a collective that shares intentions and knowledge. This stance underpins the collaborative behaviour [80] and the sharing of resources [81] and information [82] that can be observed in young human children, but not in chimpanzees.

This group-oriented stance has much in common with the we-mode. This stance involves metacognition in the sense that it takes account of the knowledge and intentions of others. However, the evidence I reviewed earlier suggests that this is an example of implicit metacognition. We adopt the group-oriented stance automatically and without awareness. This form of implicit metacognition gives a unique advantage to human interactions, but I believe that explicit metacognition endows us with even greater advantages. This is because explicit metacognition allows us to discuss aspects of our perceptual and decision-making processes with others and thereby improve our decisions

Trivially, such discussions are uniquely human in that they depend heavily on language. However, I believe that the metacognitive processes that allow the sharing of experience are also uniquely human and that they emerged before language. In the presence of this capacity for sharing, language can then arise as ‘a communicative technology’ [38,83]. But is this ability really uniquely human? Honeybees, for example, can also make joint decisions that are better than those of individuals [84]. These decisions are also made by sharing information using a primitive language: the waggle dance. Honeybees, however, can only apply their sharing skills to a small number of predetermined problems such as selecting a new nest site. Presumably, their waggle dance is an automatic rather than deliberate act, triggered by the presence of conspecifics.

Humans, as we saw in the study by Fusaroli *et al*. [51], can rapidly and flexibly develop new linguistic tools for sharing experiences when working together to solve a novel problem. In humans, the kind of collaborative behaviour seen in eusocial insects has re-evolved, but in the context of far richer and more complex underlying cognitive abilities. Indeed, Seeley and co-workers have suggested that the mechanisms that enable a swarm of bees to make complex decisions closely resemble the mechanisms by which neurons enable the primate brain to make complex decisions [85,86]. Thus, when humans make joint decisions, a whole additional layer of cognitive complexity is added.

## 9. What is explicit metacognition good for?

What are the special advantages conferred by explicit metacognition? My suggestion is that explicit metacognition enables us to share our experiences of action and sensation with others. This allows us to make joint decisions that are potentially better than those the best of us can achieve on our own [48]. Sharing experiences also enables us to develop more accurate explicit models of the world even without any objective feedback [52]. In addition, as a result of sharing experiences, we can improve our individual perception of the world [56] and alter our understanding and experience of how we make decisions.

As I pointed out at the beginning of this paper, there is a major problem with the content of explicit metacognition. First, there is the problem that we have no direct awareness of our own cognitive processes [8]. Second, and in spite of this first problem, we have no qualms in describing our cognitive processes and the outcomes of these processes, even though such descriptions often do not correspond to reality [9].

I speculate that, at the beginning of our life, the content of explicit metacognition is a blank slate on which we learn to write our experiences. And what we learn to write there is determined largely by social interactions: discussions with others, hearing stories and looking at pictures. In this way, humans develop shared views of the world and of themselves, which develop within each lifetime and which evolve across generations to form cultural norms and beliefs [87,88]. The experience of being a rational agent is one such effect of cultural norms, since claiming to be rational is one of the best ways of justifying our behaviour [89]. This development is possible precisely because of the two problems listed earlier. Since there is no direct contact with our own cognitive processes, the contents of explicit metacognition are extremely responsive to social factors, but kept within reasonable bounds by our need to interact with the physical world. Working together, we have the potential to create explicit models of our physical and our mental world that are increasingly accurate.
